# The Role of Phosphodiesterase-5 Enzyme Inhibitors and Prostacyclin Analogs in Reactive Pulmonary Vasculopathy in Systolic Heart Failure: A Narrative Review

**DOI:** 10.7759/cureus.91665

**Published:** 2025-09-05

**Authors:** Hadassa Evangeline Sekharamahanti, Dhruvil Vinaybhai Patel, Manthan Prajapati, Vaishnavi Sirekulam, Priyank Shah, Hamna Talha Peracha, Smitkumar Patel, Karthik Sai Makineni, Jennifer I Anene, Rachana Harish, Anna M Mansfield

**Affiliations:** 1 Internal Medicine, Mercy Catholic Medical Center, Philadelphia, Philadelphia, USA; 2 Internal Medicine, GMERS (Gujarat Medical Education and Research Society) Medical College, Gandhinagar, IND; 3 Medicine and Surgery, GMERS (Gujarat Medical Education and Research Society) Medical College, Gandhinagar, IND; 4 Medicine, Ballari Medical College and Research Centre (formerly Vijayanagar Institute of Medical Sciences), Ballari, IND; 5 Internal Medicine, Shiv Hospital, Patdi, IND; 6 Internal Medicine, Fatima Jinnah Medical University, Lahore, PAK; 7 Medical School, SRM Medical College Hospital and Research Centre, Chennai, IND; 8 Medicine, Ivano-Frankivsk National Medical University, Ivano-Frankivsk, UKR; 9 Internal Medicine, Bangalore Medical College and Research Institute, Bangalore, IND; 10 Medical School, Saint James School of Medicine, Arnos Vale, VCT

**Keywords:** pde-5 inhibitors, phosphodiesterase-5 inhibitors, prostacyclin analogs, pulmonary hypertension, pulmonary vasoconstriction, reactive pulmonary vasculopathy, systolic heart failure

## Abstract

Reactive pulmonary vasculopathy, the precapillary component of pulmonary hypertension due to left heart disease, is characterized by mean pulmonary arterial pressure >25 mmHg, pulmonary capillary wedge pressure >15 mmHg, transpulmonary gradient ≥12 mmHg, and pulmonary vascular resistance ≥3 Wood units. This condition complicates systolic heart failure and is associated with adverse outcomes. This narrative review evaluates the role of phosphodiesterase-5 (PDE-5) inhibitors and prostacyclin analogs in its management. A comprehensive literature search of PubMed, Cochrane Library, and Embase from January 2000 to September 2024, supplemented by reference screening, identified randomized controlled trials, observational studies, and case series investigating these therapies in systolic heart failure with pulmonary hypertension. Evidence synthesis considered both hemodynamic outcomes, such as pulmonary pressures, vascular resistance, and right ventricular function, as well as clinical endpoints, including exercise capacity, quality of life, and survival. PDE-5 inhibitors, particularly sildenafil, demonstrated improvements in pulmonary hemodynamics, endothelial function, and exercise tolerance, while prostacyclin analogs such as epoprostenol, iloprost, and treprostinil provided potent vasodilatory and antiproliferative effects with benefits in advanced disease and right ventricular protection. Combination therapy has shown potential synergistic effects, although data remain limited. Safety profiles, including risks of systemic hypotension and drug-specific side effects, are discussed separately from efficacy findings. Current evidence is limited by small study sizes, heterogeneity of design, and a lack of robust data specifically targeting Group 2 pulmonary hypertension. Overall, PDE-5 inhibitors and prostacyclin analogs appear promising in addressing reactive pulmonary vasculopathy in systolic heart failure, but larger, well-designed trials are required to establish their clinical utility.

## Introduction and background

Heart failure is broadly classified according to left ventricular ejection fraction (LVEF). Heart failure with reduced ejection fraction (HFrEF) is defined as LVEF <40%, while heart failure with preserved ejection fraction (HFpEF) is typically defined as LVEF ≥50%. An intermediate group with LVEF 41-49%, termed heart failure with mildly reduced ejection fraction (HFmrEF), is recognized in contemporary guidelines [1]. Common causes of HFrEF include myocardial infarction, dilated cardiomyopathy, valvular disease, and long-standing uncontrolled hypertension, with characteristic structural changes of eccentric hypertrophy and chamber dilation [2]. In contrast, HFpEF is more often associated with concentric remodeling, systemic hypertension, obesity, and metabolic dysfunction, reflecting distinct underlying pathophysiology [3].

Pulmonary hypertension (PH) is a frequent and clinically important consequence of left heart disease, irrespective of ejection fraction. The 2022 European Society of Cardiology/European Respiratory Society (ESC/ERS) guidelines define PH as a mean pulmonary arterial pressure (mPAP) >20 mmHg at rest, pulmonary artery wedge pressure >15 mmHg, and pulmonary vascular resistance (PVR) >2 Wood units (WU) [4]. PH due to left heart disease is classified as World Health Organization (WHO) Group 2 PH. Within this group, “reactive pulmonary vasculopathy” is often used to describe a precapillary component characterized by elevated transpulmonary gradient (TPG) and PVR, reflecting vasoconstriction and structural remodeling of the pulmonary arterioles in addition to passive backward transmission of elevated left atrial pressures. This is conceptually distinct from combined post- and pre-capillary PH (CpcPH), which is defined hemodynamically by elevated mPAP and PCWP with PVR >2 WU, but emphasizes the coexistence of both postcapillary congestion and a precapillary component [5].

Epidemiologically, reactive pulmonary vasculopathy is observed in up to 20-30% of patients with advanced HFrEF undergoing right heart catheterization, and its presence is associated with markedly increased morbidity and mortality. One cohort reported six-month mortality rising from 8.6% in HFrEF patients without PH to nearly 50% in those with reactive pulmonary vasculopathy [6]. This highlights its major clinical relevance, particularly in the context of transplant eligibility and mechanical circulatory support candidacy.

Therapeutic options for targeting the precapillary component of PH in left heart disease remain poorly defined. Phosphodiesterase-5 (PDE-5) inhibitors, primarily sildenafil, have been investigated for their potential to improve pulmonary hemodynamics, exercise tolerance, and right ventricular function in HFrEF. However, results are conflicting: small single-center studies have suggested improvements in exercise capacity and PVR [7], while larger randomized controlled trials (RCTs), such as the RELAX trial in HFpEF, failed to show significant benefit [8]. Prostacyclin analogs, including epoprostenol, iloprost, and treprostinil, are potent vasodilators with antiproliferative and antifibrotic properties, and they may offer benefit in advanced reactive vasculopathy by addressing pulmonary vascular remodeling more directly [9]. Importantly, these therapies are more biologically relevant to HFrEF than to HFpEF, since HFrEF is characterized by progressive right ventricular-pulmonary vascular uncoupling, which amplifies the impact of precapillary vasoconstriction and remodeling. Taken together, PDE-5 inhibitors and prostacyclin analogs represent promising, though controversial, strategies for managing reactive pulmonary vasculopathy in systolic heart failure. Their role remains uncertain due to heterogeneity of study populations, inconsistent trial results, and lack of definitive large-scale evidence, underscoring the need for further investigation.

## Review

This narrative review synthesizes current evidence regarding the role of PDE-5 inhibitors and PGI₂ analogs in managing reactive pulmonary vasculopathy associated with systolic heart failure. Relevant literature from January 2000 to September 2024 was identified through searches of PubMed, Cochrane Library, and Embase, supplemented by references from key articles. Search terms included “phosphodiesterase-5 inhibitors”, “prostacyclin analogs”, “reactive pulmonary vasculopathy”, and “systolic heart failure”. Various study designs, including RCTs, observational studies, and pertinent case series, were considered to capture the breadth of available evidence. The discussion integrates findings to highlight therapeutic effects, safety considerations, and gaps in knowledge, aiming to provide a comprehensive and clinically relevant overview.

The management of heart failure has evolved significantly with the introduction of pharmacological agents, among which PDE-5 inhibitors and PGI₂ analogs have gained prominence. These drugs exert effects through distinct yet complementary mechanisms, ultimately influencing vascular tone and cardiac function.

PDE-5 inhibitors, such as sildenafil and tadalafil, primarily function by inhibiting the degradation of cyclic guanosine monophosphate (cGMP), a crucial signaling molecule that promotes vasodilation. By preventing cGMP breakdown, these inhibitors increase its levels, leading to smooth muscle relaxation and vasodilation in pulmonary and systemic circulation. PDE-5 inhibition, particularly via sildenafil, has shown therapeutic benefits in heart failure, targeting both pulmonary and systemic circulations [10]. Pulmonary vasoconstriction, a key feature of heart failure, is alleviated as sildenafil preferentially lowers pulmonary vascular pressure and resistance without significantly affecting systemic arterial or pulmonary wedge pressures. Studies have demonstrated that sildenafil improves pulmonary hemodynamics at rest and during exercise, with long-term efficacy in reducing pulmonary pressures [11].

Sildenafil also improves lung diffusion capacity for carbon monoxide by over 10%, particularly increasing alveolar-capillary membrane conductance, suggesting involvement of nitric oxide and cGMP pathways. Systemic effects include reduced systemic vascular resistance, arterial stiffness, and improved endothelial function. In patients with heart failure, sildenafil decreases augmentation index and improves flow-mediated dilation, with doses as low as 25 mg effective. Combined use of sildenafil and angiotensin-converting enzyme (ACE) inhibitors, such as ramipril, further enhances endothelial benefits [12,13].

Experimental and early clinical studies suggest that sildenafil may attenuate adverse cardiac remodeling through cGMP-mediated mechanisms. In preclinical models, sildenafil has been shown to reverse hypertrophy in pressure-overloaded hearts, protect against myocyte apoptosis, and mitigate ischemia-reperfusion injury, including doxorubicin-induced cardiotoxicity. Small studies in human myocardium have also indicated potential benefits in reducing apoptosis and preserving contractility in hypertrophied right ventricles following myocardial infarction [13]. However, these findings remain largely investigational, and the therapeutic role of sildenafil in preventing or reversing cardiac remodeling in heart failure has not yet been established in large-scale clinical trials.

Sildenafil improves exercise capacity in heart failure patients by increasing peak oxygen consumption (VO2), improving right ventricular function, and enhancing gas exchange during exercise. It also modulates ventilatory response to carbon dioxide (VE/VCO2), improving exercise ventilatory efficiency by reducing dead space ventilation and promoting better muscle perfusion. These findings suggest a broad therapeutic role for PDE-5 inhibitors in heart failure management [10,14].

PGI₂ analogs, particularly epoprostenol and treprostinil, play a crucial role in managing pulmonary vasculopathy and heart failure outcomes, especially in pulmonary arterial hypertension (PAH). These synthetic derivatives of PGI₂ are potent vasodilators that also inhibit platelet aggregation. Their therapeutic effects are mediated through activation of PGI₂ receptors, leading to vasodilation of pulmonary and systemic arterial beds, reducing PVR, and improving cardiac output [15].

PGI₂ is produced by endothelial cells and maintains vascular homeostasis by promoting vasodilation and inhibiting platelet aggregation [16]. In patients with PAH, there is a significant imbalance between vasodilators like PGI₂ and vasoconstrictors such as thromboxane A2, leading to increased PVR and right heart failure [17]. PGI₂ analogs, such as iloprost and treprostinil, mimic endogenous PGI₂, restoring this balance and improving hemodynamics. These agents effectively dilate the pulmonary arterial bed, alleviating elevated pressures characteristic of PAH [15,17].

Beyond vasodilation, PGI₂ analogs exert direct cardioprotective effects on the right ventricle (RV), reducing collagen deposition and fibrosis often worsened by hemodynamic overload in PAH. This dual role alleviates PVR while protecting cardiac function [18].

Combination therapy with PDE-5 inhibitors and PGI₂ analogs has been explored, revealing potential synergistic effects that enhance therapeutic outcomes. Concurrent use may allow lower PGI₂ doses, reducing adverse effects associated with higher doses [19]. Meta-analyses indicate that combination therapy improves clinical outcomes in people with PAH, although optimal regimens require further study [20]. PDE-5 inhibitors augment PGI₂ effects by increasing cGMP levels, further promoting vasodilation and cardiac output [21].

Despite benefits, both PDE-5 inhibitors and PGI₂ analogs have limitations. PDE-5 inhibitors can cause systemic hypotension, especially when combined with nitrates, posing risks for heart failure patients who may be hemodynamically compromised [22]. Long-term safety in heart failure populations remains under investigation, with some studies suggesting protective effects against ischemic events and disease progression [23]. However, evidence is inconclusive, warranting further research.

Summary of efficacy and safety of PDE-5 inhibitors and PGI₂ analogs

Several FDA-approved PDE-5 inhibitors exist, including sildenafil, vardenafil, tadalafil, and avanafil [24]. These drugs are indicated for idiopathic PH and heart failure, among other conditions. PH is classified into five subgroups: PAH, PH due to left-sided heart disease, PH due to chronic lung disease, chronic thromboembolic PH (CTEPH), and PH with unclear or multifactorial mechanisms [25]. PDE-5 inhibitors are recommended as first-line treatment for PAH. He et al. summarized six studies involving 729 patients treated with PDE-5 inhibitors, showing a 71% reduction in mortality and increased six-minute walk distance (6MWD) [25]. These studies used sildenafil, tadalafil, and vardenafil, demonstrating improved hemodynamics and reduced clinical worsening in patients with PAH.

The RELAX study by the Heart Failure Clinical Research Network was the first multicenter trial assessing PDE-5 inhibitors in heart failure with preserved ejection fraction (HFpEF; EF ≥50%) [8, 26]. The sildenafil-treated group showed no improvement in exercise capacity measured by VO2 [27]. Conversely, an open-label pilot study with 30 patients receiving sildenafil reported a 50 m increase in 6MWD and significant improvement in New York Heart Association (NYHA) functional class and exercise capacity [28].

PGI₂ analogs are used primarily to treat moderate and advanced PAH, categorized as NYHA functional classes II to IV [29]. FDA-approved PGI₂ analogs, epoprostenol, treprostinil, and iloprost, promote vasorelaxation and suppress vascular smooth muscle proliferation [29]. Epoprostenol is preferred due to its significant improvements in 6MWD, functional class, and all-cause mortality [29]. However, the efficacy and safety of these drugs in PAH require ongoing evaluation.

Clinical significance of the findings

Despite available therapies, PAH remains fatal [30]. PDE-5 inhibitors are mainly used for mild PAH, while PGI₂ analogs are indicated for moderate to severe disease. The findings of the Ambrisentan and Tadalafil in Patients with Pulmonary Arterial Hypertension (AMBITION) trial support a combination therapy with ambrisentan (an endothelin receptor antagonist) and tadalafil (a PDE-5 inhibitor), demonstrating improved 6MWD and long-term outcomes in patients with PAH [30]. A systematic review by Bisserier et al., including 10 trials with 1,828 patients treated with PGI₂ analogs, found significant improvement in clinical worsening [31]. Evidence for PDE-5 inhibitors in patients with HFpEF and combined post- and pre-capillary PH (CpcPH) remains limited, and further randomized trials are required to clarify their role in this population.

Common and Serious Adverse Effects

The safety profile of PDE-5 inhibitors is generally well characterized, with common adverse effects including headache, flushing, nasal congestion, dyspepsia, dizziness, and visual disturbances [32]. Less frequent but clinically important reactions include hypotension, rhinitis, back pain, myalgias, and, rarely, hearing loss or non-arteritic anterior ischemic optic neuropathy. Associations with melanoma and prostate cancer have been reported in observational studies, but the evidence remains limited and controversial, without consistent confirmation in randomized trials [33]. Importantly, most safety data are derived from patients with erectile dysfunction or PAH, with limited direct evidence in heart failure populations [34].

PGI₂ analogs are associated with headache, jaw pain, nausea, vomiting, diarrhea, flushing, extremity pain, cough, and thrombocytopenia [35]. In clinical practice, PGI₂ therapy carries additional risks related to delivery method, including catheter-related infections and infusion-site reactions with continuous intravenous or subcutaneous administration. These practical complications are particularly relevant for patients with heart failure, who may already be vulnerable to infection and hemodynamic instability.

Comparative Safety Profiles

A meta-analysis of PAH therapies reported that tadalafil (40 mg) combined with ambrisentan (10 mg) was associated with the greatest improvement in 6MWD, followed by epoprostenol and high-dose intravenous treprostinil [36]. Other agents, including sildenafil, bosentan, riociguat, and iloprost, also demonstrated improvements compared with placebo, although these were not uniformly statistically significant, and the clinical meaningfulness of some changes in 6MWD remains debated. Importantly, these data are derived from PAH cohorts, and the benefits and risks of such therapies cannot be directly extrapolated to patients with Group 2 PH or HFrEF, where pathophysiology and treatment responses may differ.

Practical recommendations for clinical practice

The 2022 European Society of Cardiology/European Respiratory Society (ESC/ERS) guidelines emphasize optimizing management of underlying left heart disease before considering PH-specific therapies [37]. Crucially, drugs approved for PAH, including PDE-5 inhibitors and PGI₂ analogs, are not recommended for PH due to left heart disease, given the lack of consistent benefit and potential for harm. FDA-approved PGI₂ analogs (epoprostenol, treprostinil, and iloprost) are restricted to WHO Group 1 PH [30]. Standard heart failure therapies such as ACE inhibitors, ARBs, ARNIs, and beta-blockers are not indicated in PAH unless required for concomitant cardiovascular conditions [37].

Emerging therapies remain under investigation exclusively in PAH. For example, sotatercept significantly improved 6MWD at 24 weeks in symptomatic PAH patients in the STELLAR (Sotatercept for the Treatment of Pulmonary Arterial Hypertension) trial [38], but its efficacy and safety in heart failure-related PH remain unstudied. Additional investigational strategies include anticoagulation, recombinant fusion proteins, stem cell therapies, and the potential use of sodium-glucose cotransporter 2 (SGLT2) inhibitors to mitigate vascular stiffening [39].

For patients with left heart disease who are considered for off-label use of PAH-targeted therapies, careful monitoring for systemic hypotension, arrhythmias, renal dysfunction, and infection risk is essential. Until adequately powered RCTs are available, the use of PDE-5 inhibitors or PGI₂ analogs in HFrEF with reactive pulmonary vasculopathy should remain restricted to clinical research or highly selected circumstances.

## Conclusions

The role of PDE-5 inhibitors and PGI₂ analogs in reactive pulmonary vasculopathy associated with systolic heart failure remains investigational. Evidence is limited, often extrapolated from PAH studies, and trial outcomes in Group 2 PH have been inconsistent, with several negative or inconclusive results. These agents are not guideline-recommended for heart failure-related PH, and their clinical use should therefore be considered experimental. Long-term safety data are lacking, and practical barriers, such as the invasive administration routes required for some PGI₂ analogs, further limit applicability. Large, well-designed RCTs in patients with HFrEF and reactive pulmonary vasculopathy are urgently needed to clarify efficacy, safety, and appropriate patient selection. Until then, their use should remain restricted to research or highly selected clinical scenarios.
